# Characterization of Retinal Structure in *ATF6*-Associated Achromatopsia

**DOI:** 10.1167/iovs.19-27047

**Published:** 2019-06

**Authors:** Rebecca R. Mastey, Michalis Georgiou, Christopher S. Langlo, Angelos Kalitzeos, Emily J. Patterson, Thomas Kane, Navjit Singh, Ajoy Vincent, Anthony T. Moore, Stephen H. Tsang, Jonathan H. Lin, Marielle P. Young, M. Elizabeth Hartnett, Elise Héon, Susanne Kohl, Michel Michaelides, Joseph Carroll

**Affiliations:** 1 Department of Ophthalmology & Visual Sciences, Medical College of Wisconsin, Milwaukee, Wisconsin, United States; 2 UCL Institute of Ophthalmology, University College London, London, United Kingdom; 3 Moorfields Eye Hospital, London, United Kingdom; 4 Department of Cell Biology, Neurobiology & Anatomy, Medical College of Wisconsin, Milwaukee, Wisconsin, United States; 5 Department of Ophthalmology and Vision Sciences, The Hospital for Sick Children, The University of Toronto, Toronto, Canada; 6 Department of Ophthalmology, University of California-San Francisco Medical School, San Francisco, California, United States; 7 Jonas Children's Vision Care, Departments of Ophthalmology, Pathology and Cell Biology, Columbia Stem Cell Initiative, New York, New York, United States; 8 Department of Ophthalmology, University of California-San Diego, La Jolla, California, United States; 9 Department of Pathology, University of California-San Diego, La Jolla, California, United States; 10 Moran Eye Center, University of Utah, Salt Lake City, Utah, United States; 11 Centre for Ophthalmology Institute for Ophthalmic Research, University of Tübingen, Tübingen, Germany

**Keywords:** *ATF6*, achromatopsia, foveal hypoplasia, cones

## Abstract

**Purpose:**

Mutations in six genes have been associated with achromatopsia (ACHM): *CNGA3*, *CNGB3*, *PDE6H*, *PDE6C*, *GNAT2*, and *ATF6. ATF6* is the most recent gene to be identified, though thorough phenotyping of this genetic subtype is lacking. Here, we sought to test the hypothesis that *ATF6*-associated ACHM is a structurally distinct form of congenital ACHM.

**Methods:**

Seven genetically confirmed subjects from five nonconsanguineous families were recruited. Foveal hypoplasia and the integrity of the ellipsoid zone (EZ) band (a.k.a., IS/OS) were graded from optical coherence tomography (OCT) images. Images of the photoreceptor mosaic were acquired using confocal and nonconfocal split-detection adaptive optics scanning light ophthalmoscopy (AOSLO). Parafoveal cone and rod density values were calculated and compared to published normative data as well as data from two subjects harboring *CNGA3* or *CNGB3* mutations who were recruited for comparative purposes. Additionally, nonconfocal dark-field AOSLO images of the retinal pigment epithelium were obtained, with quantitative analysis performed in one subject with *ATF6*-ACHM.

**Results:**

Foveal hypoplasia was observed in all subjects with *ATF6* mutations. Absence of the EZ band within the foveal region (grade 3) or appearance of a hyporeflective zone (grade 4) was seen in all subjects with *ATF6* using OCT. There was no evidence of remnant foveal cone structure using confocal AOSLO, although sporadic cone-like structures were seen in nonconfocal split-detection AOSLO. There was a lack of cone structure in the parafovea, in direct contrast to previous reports.

**Conclusions:**

Our data demonstrate a near absence of cone structure in subjects harboring *ATF6* mutations. This implicates *ATF6* as having a major role in cone development and suggests that at least a subset of subjects with *ATF6-*ACHM have markedly fewer cellular targets for cone-directed gene therapies than do subjects with *CNGA3*- or *CNGB3*-ACHM.

Achromatopsia (ACHM) is an autosomal recessive condition that is characterized by a lack of cone photoreceptor function. Subjects present at birth or early infancy with nystagmus, reduced visual acuity, photoaversion, and reduced or absent color vision.^1^,^2^ Disease-causing sequence variants in the genes encoding the alpha and beta subunits of the cone-specific cyclic nucleotide gated ion channel (*CNGA3* and *CNGB3*, respectively) account for approximately 70% of all cases of ACHM,^3^ although variants in genes encoding for other components of the cone phototransduction pathway (*GNAT2*, *PDE6H*, *PDE6C*)^4^–^6^ have also been associated with ACHM. Advances in genetic testing have resulted in discovery of a genetic basis for nearly all patients with ACHM.^7^ Any remaining molecularly unconfirmed cases of ACHM may actually be misdiagnosed cases of atypical cone-rod dystrophy. That said, additional genes cannot be ruled out. For example, mutations in *ATF6* were recently identified in some subjects with ACHM who were negative for mutations in the aforementioned phototransduction genes.^8^–^11^ The ATF6 protein encodes an endoplasmic reticulum (ER) localized transcription factor that helps maintain ER homeostasis, as part of the unfolded protein response (UPR). As one of three transmembrane proteins that regulate the UPR, ATF6 is activated upon ER stress to transcriptionally upregulate ER chaperones and ER protein folding enzymes that help alleviate ER stress and restore cellular homeostasis.^12^–^14^ Disease-causing sequence variants in *ATF6* result in dysfunction of this critical signaling pathway. This seems especially detrimental to photoreceptor cells, which are among the most metabolically active cells in the human body.^15^,^16^

As gene replacement therapy efforts in ACHM target cone photoreceptors, it is important to fully understand how these cones are affected by a given genotype. A variety of noninvasive imaging tools are available for examining retinal structure, and these have already been applied extensively to the more common forms of ACHM.^17^–^21^ Optical coherence tomography (OCT) provides visualization of retinal lamination, enabling measurements of retinal layer thickness and intensity. Of particular interest are the hyperreflective (ellipsoid zone, EZ, and interdigitation zone, IZ) and hyporeflective (outer nuclear layer, ONL) bands associated with the photoreceptors.^22^,^23^ In subjects with *CNGA3*- or *CNGB3*-associated ACHM (abbreviated *CNGA3*-ACHM and *CNGB3*-ACHM, respectively) the EZ band at the fovea is disrupted or absent in approximately 68% of cases.^20^,^21^ The thickness of the ONL is also significantly reduced in *CNGA3*- and *CNGB3*-ACHM, although there is substantial variability among subjects.^20^,^21^,^24^ Another imaging tool—adaptive optics scanning light ophthalmoscopy (AOSLO)—enables noninvasive, cellular resolution imaging of the rod and cone photoreceptor mosaic. In subjects with *CNGA3*- or *CNGB3*-ACHM, there is an absence of normal waveguiding cone photoreceptors when imaged with the confocal modality of AOSLO,^17^,^21^ although the rod photoreceptors appear normal (Patterson E, et al. *IOVS* 2018;59:ARVO E-Abstract 652). Using a nonconfocal split-detection AOSLO technique, extensive remnant photoreceptor inner segment structures have been observed in these subjects that coincide spatially with the non-waveguiding cones seen in the confocal images.^21^,^25^

To date, the imaging findings in *ATF6*-associated ACHM (*ATF6*-ACHM) include bilateral loss of the foveal reflex on fundus examination,^8^ and variable abnormalities in fundus autofluorescence imaging.^9^ In addition, foveal hypoplasia with minimal foveal pit formation has been observed in all subjects with *ATF6-*ACHM to date.^9^,^10^ A single study used a commercial prototype AOSLO to image one subject with *ATF6-*ACHM,^9^ and reported supranormal cone density outside the central fovea. This is in stark contrast to previous reports in patients with *CNGA3*- or *CNGB3*-ACHM,^17^,^19^,^21^ raising questions about possible genotype-dependent differences in cone structure as well as highlighting the need to more fully examine cone structure in additional subjects with *ATF6-*ACHM. Here, we sought to further examine retinal structure in *ATF6*-ACHM using OCT and AOSLO.

## Methods

### Subjects

Seven genetically confirmed subjects from five nonconsanguineous families (MM_0044 and MM_0043, and MM_0147 and MM_0152 are siblings, respectively) were recruited through one of four sites (Table 1). Informed consent was obtained from all participants. Three subjects were imaged at the Medical College of Wisconsin and four subjects were imaged at Moorfields Eye Hospital, London. Data from two additional non-*ATF6* ACHM subjects, one *CNGA3*-ACHM and one *CNGB3*-ACHM, were included for comparison (see Table 1 for details). This study followed the tenets of the Declaration of Helsinki and was approved by the institutional review boards at the Medical College of Wisconsin (PRO17439 and PRO30741) and University College London/Moorfields Eye Hospital (UCL/MEH).

**Table 1:** Subject Demographics

**Subject**	**Sex**	**Age**	**Gene**	**Genotype**	**Axial Length, mm**	
					**Right Eye**	**Left Eye**
JC_10069*	M	18	*CNGA3*	c.847C>T / p.Arg283Trp	23.13	23.59
				c.542A>G / p.Tyr181Cys		
JC_10232†	M	18	*CNGB3*	c.1148delC / p.Thr383Ile fs*13, homozygous	26.22	27.06
MM_0043‡	F	49	*ATF6*	c.970C>T / p.Arg324Cys, homozygous	24.33	24.38
MM_0044‡	F	44	*ATF6*	c.970C>T / p.Arg324Cys, homozygous	20.60	20.51
MM_0147‡	F	25	*ATF6*	c.1187+5G>C / p.Asn366His fs*12, homozygous	22.59	22.58
MM_0152‡	F	26	*ATF6*	c.1187+5G>C / p.Asn366His fs*12, homozygous	23.08	23.12
AV_10962	F	25	*ATF6*	c.1699T>A / p.Tyr567Asn, homozygous	22.60	22.55
TM_11446	M	10	*ATF6*	c.970C>T / p.Arg324Cys	24.42	24.51
				c.(82+1_83-2)_(247+1_248-1_del)§		
JC_11438||	F	6	*ATF6*	c.1126C>T / p.Arg376Ter	19.45	19.34
				c.1533+1G>C		

F, female; M, male.

* Subject previously reported in multiple studies.^25^,^39^

† Subject previously reported in multiple studies as ACHM-001-CEI-001.^21^,^47^

‡ Subject reported by Kohl et al.^9^

§ This is a novel deletion in exons 2 and 3, which removes part of the acidic activator domain of *ATF6* required for its transcriptional activator properties.

|| Subject reported by Xu et al.^10^

### Genetics

All seven subjects had genetic sequencing performed, with five of the subjects previously reported in the literature (Table 1).^9^,^10^ One unreported mutation was found (TM_11446) that deleted 1637 nucleotides and leads to a deletion of exons 2 and 3 of the *ATF6* gene. This deletion is in the transcription activation region within the cytosolic domain, and thus would be predicted to be deleterious (though no in silico predictions are available for this deletion). Familial testing confirmed that this deletion was in trans to the other mutation in this subject (c.970C>T / p.Arg324Cys), which has been previously reported in other patients.^9^

### OCT Imaging and Analysis

Prior to imaging, the combination of tropicamide (1%) and phenylephrine hydrochloride (2.5%) was used for cycloplegia and pupillary dilation in all but two subjects who were children, and Cyclomydril (Alcon Laboratories, Fort Worth, TX, USA) was used instead. Bioptigen SD-OCT (Leica Microsystems, Wetzlar, Germany) was used to acquire volume and horizontal line scans at the fovea. Horizontal line scans were obtained with a nominal scan length of 7 or 8 mm and volume scans were 7 × 1 and 7 × 7 mm in scan length. Images in both eyes of all subjects were acquired, with the exception of JC_11438 in whom only the right eye was imaged. The OCT images were processed using ImageJ,^26^ and between 7 and 29 individual B-scans were registered and averaged to improve signal-to-noise ratio for subsequent analysis, as previously described.^27^ Foveal line scans were graded for EZ disruption by one observer (R.R.M.) using a previously established grading system.^20^,^21^ In summary, grade 1 corresponds to a continuous EZ band, grade 2 is EZ disruption, grade 3 is the absence of the EZ band, grade 4 is the appearance of a hyporeflective zone, and grade 5 is outer retinal atrophy. Foveal ONL thickness—defined as the distance between the inner limiting membrane and external limiting membrane (ELM) in the case of complete foveal excavation or the distance between the outer plexiform layer and ELM in the case of incomplete foveal excavation^20,21^—was evaluated using OCT Reflectivity Analytics (ORA) software.^24^,^28^ Foveal hypoplasia, defined as the presence of one or more inner retinal layers through the fovea, was also assessed.^18^,^20^,^29^ Axial length measurements (IOL Master; Carl Zeiss Meditec, Dublin, CA, USA) were obtained in both eyes of all subjects for use in deriving the lateral scale of the OCT and AOSLO retinal images.

### AOSLO Imaging and Analysis

High-resolution imaging was attempted in all seven subjects using one of three previously described custom-built AOSLO devices.^25^,^30^,^31^ Videos were acquired at the fovea in addition to a strip extending from the fovea out to 10° in the temporal direction. Since the system uses optical scanners to capture videos, there is inherent distortion, which is made worse by involuntary eye movements like nystagmus seen in ACHM subjects. The AOSLO videos were registered and averaged^32^ to an automatically selected reference frame.^33^ This method increases the signal-to-noise ratio of the resulting processed images. Three AOSLO modalities (confocal, split-detection, and dark-field) are acquired simultaneously,^25^,^34^ and therefore, at the same location. The best-quality processed images were determined manually and imported into a program that automatically aligned the individual AOSLO images from each location to create a larger montage spanning the region of the retina that was imaged.^35^ Upon completion, the images were entered into Adobe Photoshop (Adobe Systems, Inc., San Jose, CA, USA) where alignment of each image was manually checked and adjusted as needed. Montages of different fields of view (ranging from 1.0° × 1.0° to 3.0° × 3.0°) were then scaled and combined into a single montage for each subject for analysis.

Two of the seven subjects had montages that extended to approximately 10° in the temporal direction with sufficient image quality to determine cell counts across the imaged region. Peak cone density is typically used to aid determination of eccentricity; however, subjects with *ATF6-*ACHM have minimal, if any, discernable cones in the fovea so peak cone density could not be calculated. The foveal center was therefore identified as the geometric center of the foveal lesion, found from marking the outermost edges in the vertical and horizontal directions. Measurements of eccentricity were referenced to this location. Regions of interest, 100 × 100 μm in size, were selected at 5° and 10° temporal to the foveal center using semiautomated cone counting software (Translational Imaging Innovations).^36^ In *ATF6*-ACHM, all objects that represented small, round structures with an approximate Gaussian reflectivity profile within the region of interest were counted in the confocal modality and used to estimate cell densities.^19^ In *CNGA3*- and *CNGB3*-ACHM the reflective structures seen in the confocal modality were representative of rod photoreceptors^17^,^21^; therefore the hyporeflective structures surrounded by hyperreflective rods were counted to derive cone density measurements at both eccentricities for comparison to the cell density counts of *ATF6*-ACHM.

### Statistics

Data analysis included statistical tests performed using Prism version 7.0 (GraphPad Software, La Jolla, CA, USA). Normality was assessed using the Shapiro-Wilk normality tests for all data sets. Nonparametric tests were used to assess nonnormally distributed data.

## Results

### Disrupted Foveal Anatomy Observed in All Subjects With *ATF6-*ACHM

**Figure 1:**
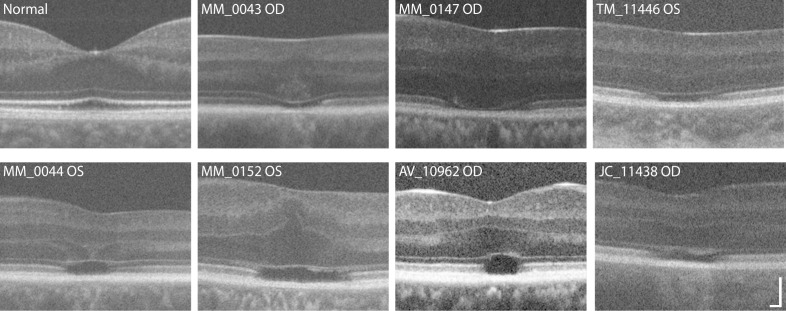
Disrupted foveal structure in ATF6-ACHM visualized with SD-OCT. An SD-OCT scan at the fovea of a subject with normal vision is shown for reference. Severe foveal hypoplasia (persistent inner retinal layers at the fovea) was observed in all seven subjects. All subjects showed disruption of the EZ, either as a grade 3 (top three subjects) or a grade 4 (bottom four subjects). In contrast, subjects with CNGA3- or CNGB3-ACHM have a more variable EZ phenotype, with all five EZ grades having been observed.^20^,^21^ Scale bar: 100 μm.

**Figure 2:**
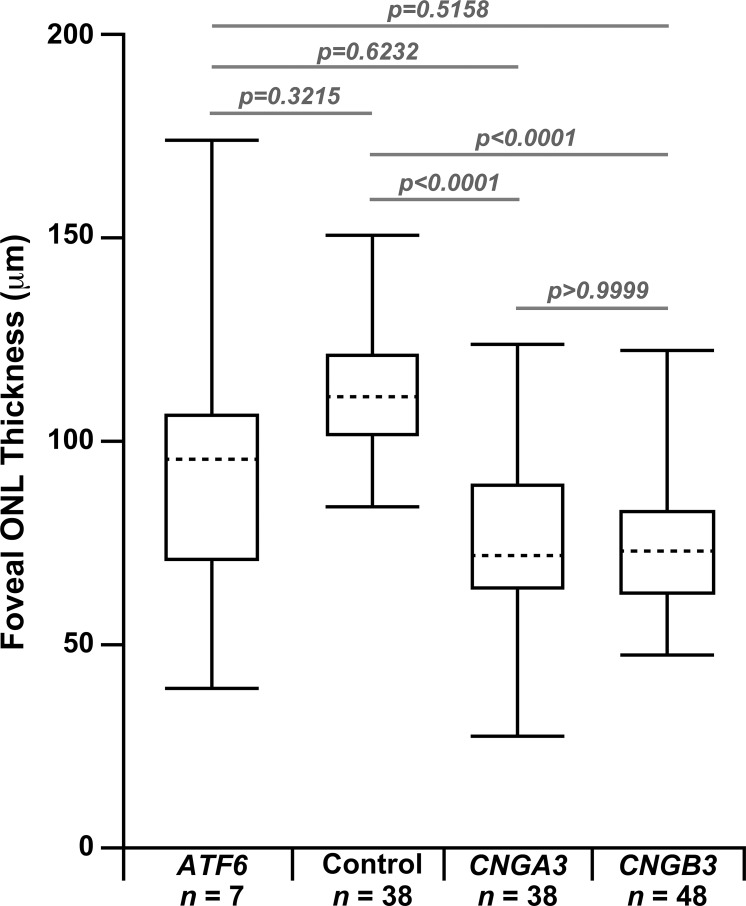
Variably reduced ONL thickness in ATF6-ACHM. Box plots show the median ONL thickness (dashed horizontal line) and 25th and 75th quartile (solid horizonal line) for each group of subjects. The error bars represent the minimum and maximum values within each group. A Kruskal-Wallis test revealed a significant difference between groups (P < 0.0001), and Dunn's multiple comparisons test was used to examine difference between specific pairs of groups. Individual P values are listed on the graph for clarity. The previously published CNGA3-39 and CNGB3-ACHM subjects^21^ have reduced ONL thickness compared to the published control data.^24^ The subjects with ATF6-ACHM had thicker ONL compared to the CNGA3 and CNGB3 data, but were not significantly different from the controls.

Foveal hypoplasia was evident in OCT images for all subjects (Fig. 1), and is consistent with previous reports of *ATF6*-ACHM.^9^ All 21 reported *ATF6*-ACHM subjects (including those reported here) show foveal hypoplasia,^9^,^10^ which is markedly different when compared to the 97/146 previously reported subjects with *CNGA3*- and *CNGB3*-ACHM (*P* < 0.0001, Fisher's exact test).^17^,^20^,^21^,^37^,^38^ While foveal hypoplasia is observed in subjects with *CNGA3*- and *CNGB3*-ACHM,^20^ the incomplete foveal development consistently seen in *ATF6*-ACHM is unusual in comparison to the other genetic causes. In addition, disrupted foveal lamination was observed in all subjects; either grade 3 (Fig. 1, top row) or grade 4 (Fig. 1, bottom row) EZ disruption.^20^ Interestingly, there was also evidence of other disorganization seen at the fovea; with hyperreflective structures of unknown origin visible within the ONL of two subjects, MM_0043 and MM_0044, and what might be Müller glial cells below the ELM in MM_0152 and JC_11438 (Fig. 1). Foveal ONL thickness was highly variable in the seven cases studied, ranging from 39.22 to 174.04 μm, and the mean ± SD foveal ONL thickness for *ATF6*-ACHM was 95.23 ± 41.88 μm. As shown in Figure 2, this was slightly lower than previously reported values for controls (110.60 ± 15.67 μm).^24^ However, due to the small number of subjects with *ATF6-*ACHM and the larger range in their ONL thickness, this was not significant (*P* = 0.3215, Dunn's multiple comparisons test). The ONL thickness in the subjects with *ATF6*-ACHM, while greater, was not significantly different from previously reported values for *CNGA3-* (74.18 ± 21.80 μm; *P* = 0.6232, Dunn's multiple comparisons test)^39^ or *CNGB3*-ACHM (74.15 ± 15.96 μm; *P* = 0.5158, Dunn's multiple comparisons test).^21^

### Central Foveal Lesions With Ambiguous Structures Seen in AOSLO

**Figure 3:**
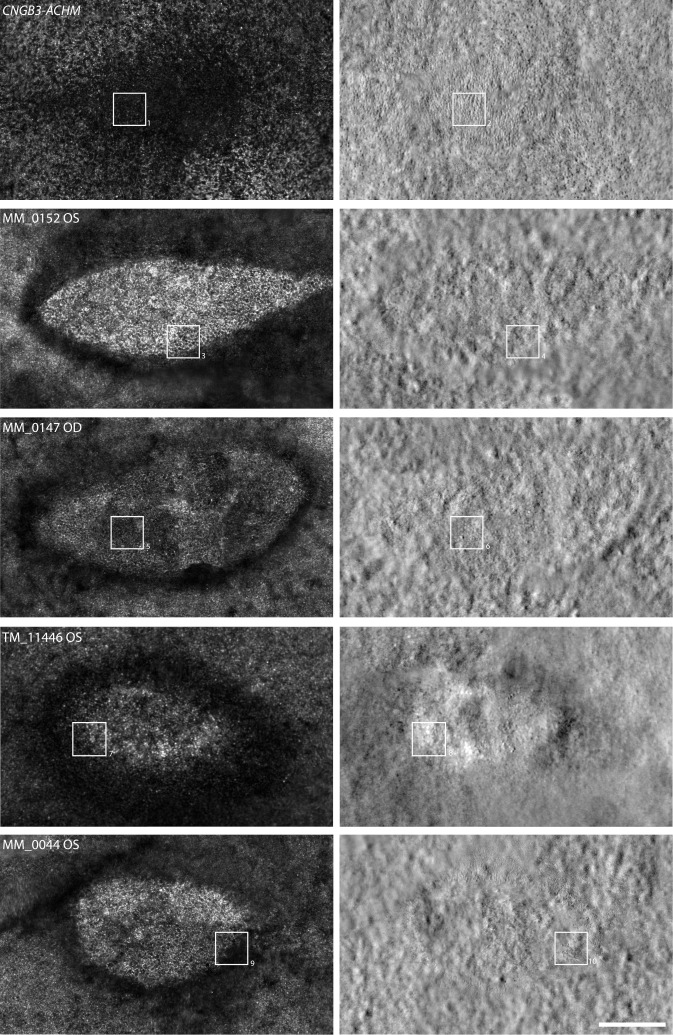
Foveal lesions in ATF6-ACHM as seen with AOSLO. Montages of the fovea are shown in both confocal (left) and split-detection (right) modalities. While subjects with CNGA3- or CNGB3-ACHM generally have a hyporeflective-appearing fovea with confocal AOSLO,^21^ the subjects with ATF6-ACHM showed an elliptical hyperreflective lesion at the fovea. The structure within this lesion was reminiscent of RPE, as has been reported in subjects with advanced cone-rod dystrophy.^57^ On split detection, the subject with CNGB3-ACHM showed numerous remnant cone structures within the foveal region, and the extent of remnant cone structure has been reported to vary across subjects by nearly 8-fold.^21^ In contrast, subjects with ATF6-ACHM had few (if any) cones visible in the split-detection images. For example, MM_0044 had a sparse array of presumed cones on the border of the foveal lesion, while MM_0147 had a few small patches of inner segments that appeared enlarged compared to the CNGB3-ACHM retina. Numbered (1–10) squares represent locations of the images shown in Figure 4. Scale bar: 200 μm.

**Figure 4:**
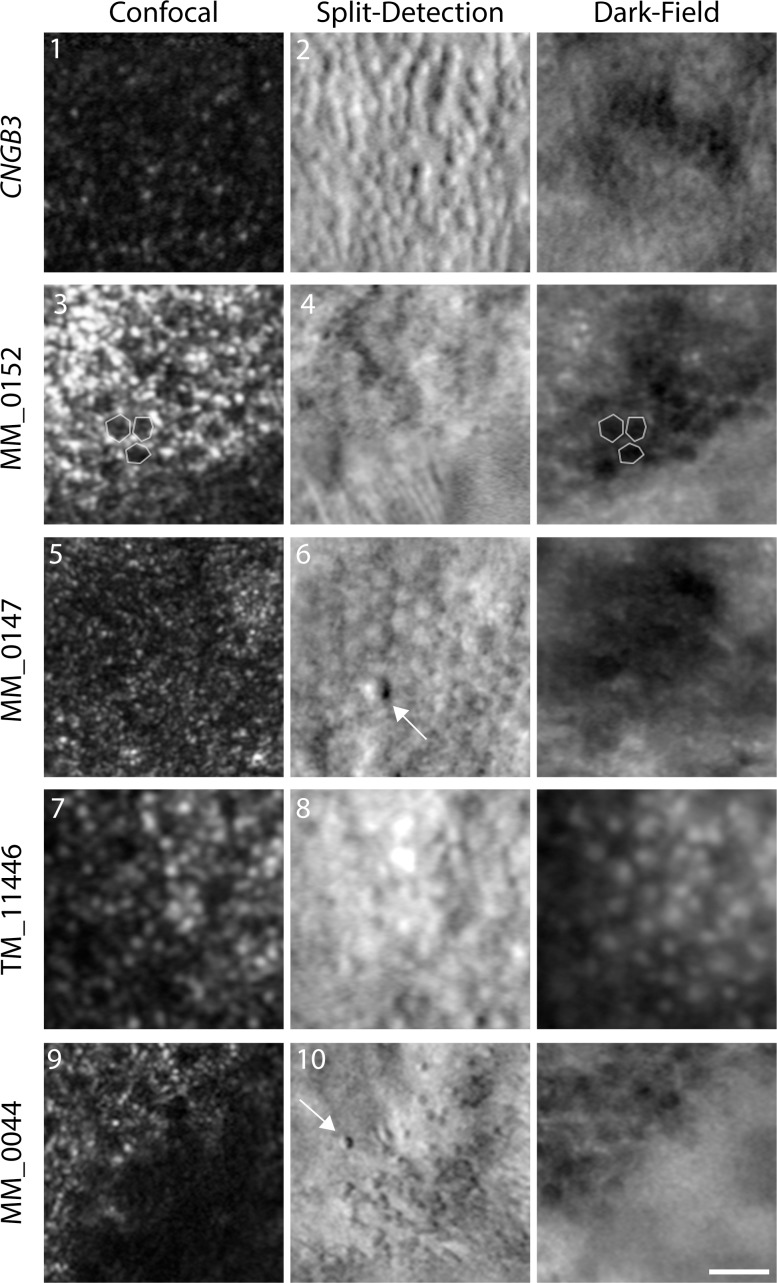
Reduced foveal cone structure in ATF6-ACHM as seen with AOSLO. Images are numbered according to the labels in Figure 3, and the three images for each subject were acquired simultaneously and are spatially coregistered. The CNGB3 image shows many remnant inner segments on split detection, though these cones are not waveguiding normally, resulting in a hyporeflective confocal image. In the subjects with ATF6-ACHM there were isolated locations of remnant cone structures observed in the split-detection images (e.g., arrows [6, 10]), though they are very different in frequency than what is typically seen in subjects with CNGA3- and CNGB3-ACHM.^21^,^39^ Within the fovea of subjects with ATF6-ACHM, the hyporeflective structure observed with confocal imaging aligned with structure in the dark-field image in many cases (see MM_0152 and MM_0044 for particularly clear examples). These images are consistent with the RPE mosaic and a general absence of cone structure (a small group of RPE cells are outlined in the confocal and dark-field images for MM_0152). One subject (TM_11446) had large reflective structures in the dark-field image, in stark contrast to the other subjects as well as previously published dark-field images.^34^ Scale bar: 25 μm.

We were not able to process or analyze the AOSLO images from two of our subjects (JC_11438 and MM_0043), due to poor image quality. However, four of the remaining five subjects had successful imaging sessions that allowed for construction of a complete foveal montage (five eyes total). Despite variations in image quality, a central foveal lesion, denoted by a distinct, dark ring in confocal AOSLO (Fig. 3), was observed in all eyes imaged. A fifth subject (AV_10962) had good image quality in both eyes, and while the presence of a foveal lesion could be confirmed, the montages were incomplete and did not include the entire lesion. Although sporadic structures were observed within all lesions using split-detector AOSLO (Figs. 3, 4), their appearance was highly variable and distinct from those seen in images of *CNGA3*- or *CNGB3*-ACHM. In the *CNGA3* and *CNGB3* forms of ACHM, a clear foveal cone mosaic can be visualized using split-detection AOSLO (Figs. 3, 4), and in some subjects the mosaic can be contiguous.^21^,^25^,^39^ Using confocal AOSLO, the majority of features at the fovea of *ATF6*-ACHM seemed to be retinal pigment epithelial (RPE) cells, observed as hyporeflective, hexagonal structures that did not align to any distinct features in the split-detection modality, but rather directly aligned with the dark-field RPE mosaic (Fig. 4, MM_0152). However, occasional isolated cone-like structures were observed in the split-detection images (Fig. 4, white arrows). Within the lesion of MM_0147, large, circular structures were visible in the nonconfocal split-detection images (Fig. 4), which could originate from swollen, non-waveguiding cones, perhaps in the process of degeneration, or from RPE cells. Additionally, the ambiguous structures congregated around the edge of the lesion in MM_0044, also seen in the nonconfocal split-detection images, could represent rods or cones (Fig. 4). These observations are in keeping with a general absence of foveal cone structure in *ATF6*-ACHM.

**Figure 5:**
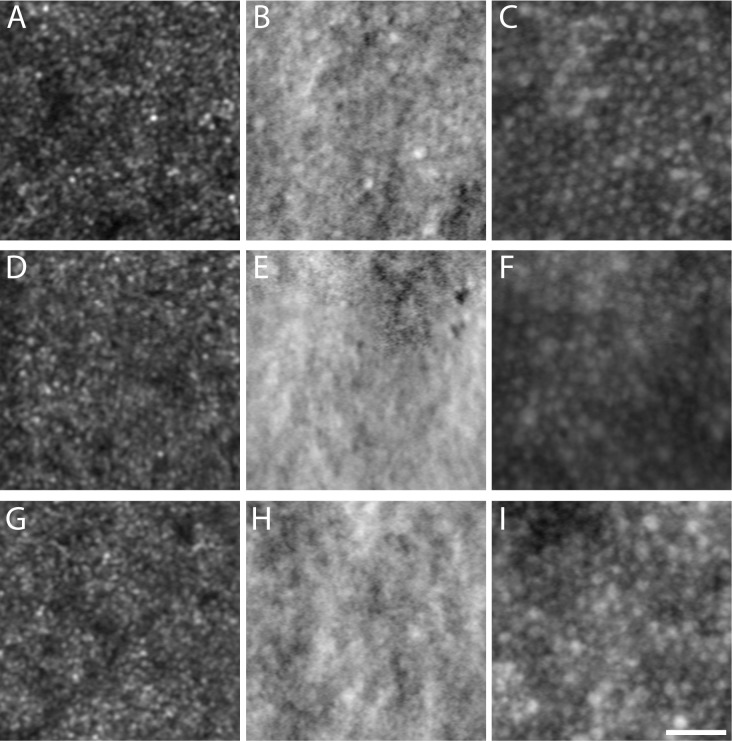
Unusual dark-field AOSLO images in a subject with ATF6-ACHM. Shown are parafoveal images from the left eye of subject TM_11446. Images were collected at 1° (A–C), 2° (D–F), or 3° (G–I) from the foveal center. Simultaneous confocal (A, D, G), split-detector (B, E, H), and dark-field images (C, F, I) were acquired at each location. The smaller reflective structures in the confocal images are likely rods, based on their small size and corresponding absence of cone inner segment structure in the split-detector images (see Figs. 3 and 6 for comparison to CNGA3- and CNGB3-ACHM). The dark-field images reveal a relatively contiguous mosaic of cell-like structures, which we propose may be RPE in origin. The densities were 5898, 5402, and 4888 cells/mm^2^ at 1°, 2°, and 3° eccentricity, respectively. These values are consistent with ex vivo and in vivo estimates of RPE cell density at similar retinal locations (see text). Scale bar: 50 μm.

Subject TM_11446 had very unusual dark-field images. Punctate structures were seen throughout the foveal region (Fig. 4) that were unlike anything we have observed in the other subjects with *ATF6*-ACHM or any previous subjects with *CNGA3*- or *CNGB3*-ACHM. We analyzed the density of these structures at three regions of interest (Fig. 5). The densities were 5898, 5402, and 4888 cells/mm^2^, at 1°, 2°, and 3° eccentricity, respectively. These values are in excellent agreement with previously reported values for RPE cell densities at similar retinal locations from histology^40^,^41^ and in vivo AOSLO imaging.^34^,^42^–^44^ While this is suggestive of an RPE origin for these structures, the appearance of the structures seen here is unlike any of the published AOSLO images of RPE cells, regardless of the modality used.

### Parafoveal Photoreceptor Mosaic in *ATF6*-ACHM Has Density Consistent With the Normal Rod, Not Cone, Mosaic

**Figure 6:**
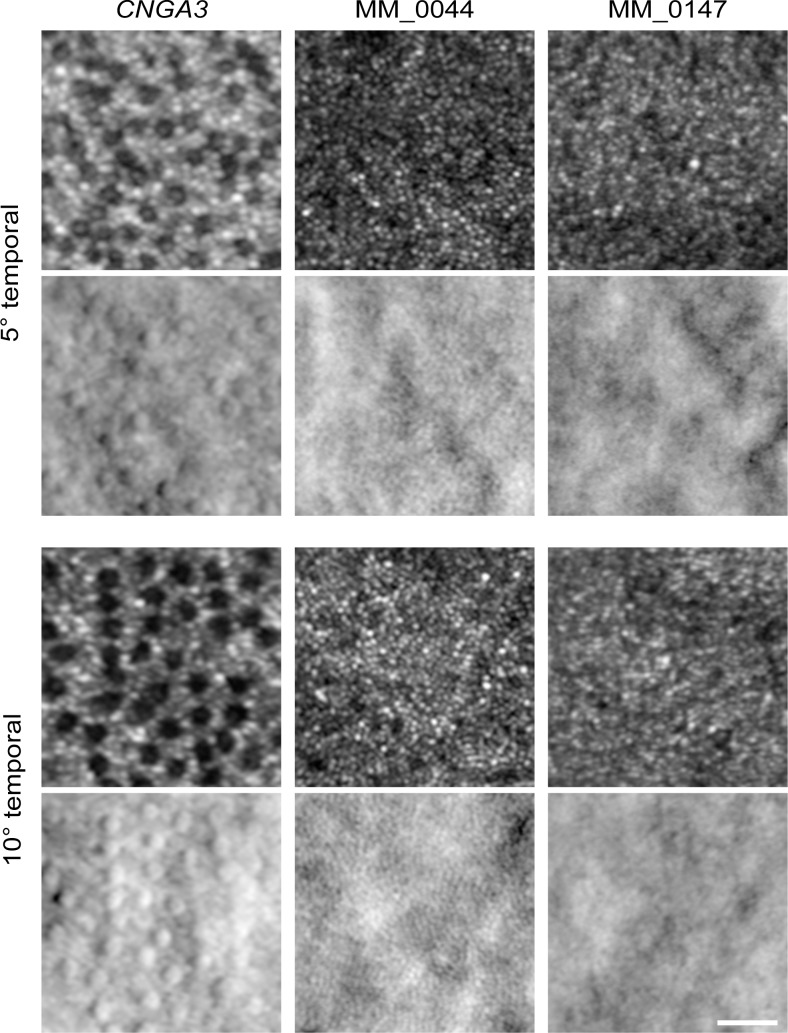
Altered parafoveal cone structure in ATF6-ACHM. In the subject with CNGA3-ACHM, the cones had reduced or absent reflectivity on confocal AOSLO (top rows at each eccentricity) but could be identified by the surrounding rod mosaic. These “dark” cones aligned with remnant inner segments seen in the corresponding split-detection image (bottom rows at each eccentricity). In contrast, subjects with ATF6-ACHM had a mosaic of uniformly sized structures on both confocal and split-detection AOSLO. These cells were much smaller than the remnant cones in the CNGA3 retina, being more similar in size to the rod photoreceptors. In addition, an analysis of cell density showed that the ATF6 parafoveal mosaic was consistent with normal rod density as opposed to cone density (see Table 2). Taken together, this suggests that subjects with ATF6-ACHM have minimal, if any, cone photoreceptors in the parafoveal region. Scale bar: 25 μm.

Two subjects (MM_0044, MM_0147) had stable fixation and good image quality that enabled reliable imaging and parafoveal analysis in the temporal retina. In the *CNGA3* and *CNGB3* forms of ACHM, both rods and cones can be easily distinguished.^21^,^25^,^39^ In these subjects, the parafoveal cones appear dark (i.e., non-waveguiding) on confocal AOSLO surrounded by the smaller hyporeflective rods, though their inner segments can be visualized in split-detection AOSLO (Fig. 6). In contrast, both *ATF6*-ACHM individuals had a contiguous mosaic of cells of uniform size on both the confocal and split-detector AOSLO images (Fig. 6). When the parafoveal cell counts of MM_0044 and MM_0147 were compared to normal rod and cone density values from imaging and histology studies at comparable eccentricities,^45^,^46^ they were more similar to normal rod density (Table 2). This is consistent with an absence of cone structure in the parafovea of these two subjects with *ATF6-*ACHM.

**Table 2:** Parafoveal Cell Counts

Subject	**Cell Density, Cells/mm^2^**	
	5°T	10°T
MM_0147	103,433	84,009
MM_0044	85,406	98,161
JC_10069 (*CNGA3*), cones	7,454	6,726
Normal rod density*	80,000	125,000
Normal cone density*	15,000	10,000

* Approximate values from prior histology^45^ and imaging studies.^46^

## Discussion

Kohl et al.^9^ suggested that a poorly formed or absent foveal pit may be a hallmark of *ATF6*-ACHM, which is confirmed by the high-resolution data presented here. Foveal hypoplasia was observed in all seven subjects with *ATF6-*ACHM, again consistent with previous reports.^9^,^10^ The increased prevalence of hypoplasia may contribute to the observed ONL differences reported in Figure 2. In addition, only grade 3 and grade 4 EZ disruption was observed in direct contrast to *CNGA3*- and *CNGB3*-ACHM, in which all five EZ grades were observed.^20^,^21^,^47^ In previous work, 2% to 15% of subjects with *CNGA3-* and *CNGB3*-ACHM had grade 3 EZ disruption and 24% to 31% had grade 4.^20^,^21^,^24^ Further disorganization was observed at the fovea including hyperreflectivity of the ONL (MM_0043, MM_0044) and apparent thickening of the ELM (MM_0152, JC_11438). These findings suggest that the normal foveal development is significantly disrupted in individuals with *ATF6-*ACHM, but it remains an unanswered question as to what aberrant development is taking place. Furthermore, nonconfocal split-detection AOSLO allowed us to observe structures in the foveal region that appear to be RPE cells from the appearance in the dark-field AOSLO, although the possibility of cones being present cannot be completely discounted. Lastly, we examined the parafoveal photoreceptor mosaic. Kohl et al.^9^ reported that individuals with *ATF6-*ACHM lacked cones at the fovea but had supranormal cone densities outside the foveal region. However, in contrast, we found a general absence of cones at the fovea and a complete absence of cones in the parafoveal regions. Although cell counts were higher in the parafovea, we hypothesize that these cells are rods rather than cones, based on both density and relative size. Commercially available AO systems, such as the one used in the earlier study,^9^ are typically geared toward more clinical use. These systems frequently have limited resolution making it difficult to disambiguate rod from cone structure and to reliably image foveal cones.^48^,^49^ As well, many prior studies erroneously assume that all reflective dots in the AO-flood or AOSLO images represent cone photoreceptors. Thus, caution should be used when interpreting such data. Nonconfocal imaging modalities like the split-detection AOSLO used here can help define remnant cone structure without being reliant on intact waveguiding properties of the cell.^25^,^50^–^52^ It is important to note that next-generation commercial systems are improving significantly, with many incorporating nonconfocal modalities,^53^–^55^ so interpretation of AOSLO images should only improve.

When the peripheral and foveal appearance on AOSLO are taken together, *ATF6*-ACHM represents a unique phenotype. The most frequently observed phenotype in *CNGA3*- and *CNGB3*-ACHM is the presence of dark, non-waveguiding cones in confocal images corresponding to remnant inner segment structures in split-detection images, albeit highly variable in density.^21^,^39^ Another AOSLO phenotype has been reported in *GNAT2*-ACHM, as these individuals have contiguous foveal cone mosaics with normal-like waveguiding behavior,^19^ consistent with reports of remnant cone function in some patients with *GNAT2* mutations.^56^ However, what has been observed here in *ATF6*-ACHM is certainly unique as there is a foveal lesion with a general absence of cones in addition to solely rod photoreceptors in the parafovea. That said, the preserved (or even increased) ONL thickness in some subjects with *ATF6-*ACHM (Fig. 2) is paradoxical given the absence of cone structure on AOSLO. This could be due to preserved cone nuclei or altered morphology of the remaining rod nuclei, or it could be an artifact caused by the severe hypoplasia.

Here, the clear visibility of the foveal RPE mosaic with confocal AOSLO in some of the subjects with *ATF6-*ACHM is also consistent with a general absence of foveal cone structure in these retinas. The RPE mosaic appearance has been reported by Scoles et al.^34^ in central serious retinopathy when the retina is detached and cross-talk between the photoreceptor layer and RPE is minimized, which in turn allows for a clear visualization of the RPE hexagonal mosaic. Furthermore, it has also been observed by Roorda et al.^57^ in cone dystrophy where the photoreceptors are eliminated, leaving the RPE visible. While dark-field AOSLO images also revealed a hexagonal RPE mosaic in some of our subjects that was similar in appearance to previously published data from multiple AOSLO modalities,^34^,^42^–^44^ the appearance of the dark-field AOSLO images in subject TM_11446 is unlike anything we have seen in imaging hundreds of patients with inherited retinal degenerations. It is difficult to speculate why these presumed RPE cells are so different in appearance or whether this has anything to do with the specific (and unique) *ATF6* mutation in this subject. While we cannot confirm the cellular identity of these unusual structures, further imaging of this patient using ICG-AOSLO,^43^ NIRAF- or SWAF-AOSLO,^44^ or even AO-OCT^58^,^59^ may aid in interpretation.

There are important limitations to the present study. First, the parafoveal data demonstrating a lack of cone structure are based on data from just two of our subjects, and the qualitative central foveal analysis was based on just five of our subjects. As such, whether these observations are generalizable to all subjects with *ATF6*-ACHM remains unknown. That said, the striking difference we observed is consistent with such an interpretation. More subjects should be examined to assess the full range of retinal/photoreceptor structure associated with these mutations. It may also be of interest to use AOSLO to examine individuals with *ATF6*-associated cone-rod dystrophy^11^ to look for cellular-resolution phenotypic differences with other forms of cone-rod dystrophy. Furthermore, the in vivo nature of these studies precludes definitive identification of the cellular structures in the images; however, we believe that the interpretation we have provided represents the most likely scenario.

This is the first in-depth analysis of retinal architecture in *ATF6*-ACHM, which is molecularly different from the other five cone phototransduction-related genetic forms of ACHM, and so it should not be surprising that the retinal phenotype starkly varies from the other forms. However, one of the more puzzling aspects of this condition is why the defect appears to be specific to cones, given the ubiquitous expression of *ATF6*. The similarity of phenotype across the subjects of varying ages supports the cone defect as being congenital and not progressive, though longitudinal studies are needed to confirm this. The absence (or severe reduction) of cone structure in *ATF6*-ACHM suggests that these patients may not respond positively to cone-directed gene replacement therapy. If there is a sparse array of remnant cones in some subjects, additional strategies involving ATF6-activating small molecules could be explored,^60^–^62^ as current methods of gene delivery via subretinal injection likely result in some photoreceptor damage. It seems more likely that alternative approaches, such as cell replacement therapy, may be worth pursuing for these patients. The ability to quantify cone structure on an individualized basis should be valuable for selecting the best approach for a given patient as well as monitoring structural changes following intervention.
